# Seroprevalence of Hepatitis E Virus in Moose (*Alces alces*), Reindeer (*Rangifer tarandus*), Red Deer (*Cervus elaphus*), Roe Deer (*Capreolus capreolus*), and Muskoxen (*Ovibos moschatus*) from Norway

**DOI:** 10.3390/v13020224

**Published:** 2021-02-01

**Authors:** Carlos Sacristán, Knut Madslien, Irene Sacristán, Siv Klevar, Carlos G. das Neves

**Affiliations:** 1 Norwegian Veterinary Institute, P.O. Box 750, NO-0106 Oslo, Norway; knut.madslien@vetinst.no (K.M.); siv.klevar@vetinst.no (S.K.); 2 Facultad de Ciencias de la Vida, Universidad Andres Bello, República 252, 8320000 Santiago, Chile; isacristan.vet@gmail.com

**Keywords:** emerging infectious diseases, cervids, One Health, *Orthohepevirus*, ungulates, viral hepatitis, wildlife, zoonosis

## Abstract

Hepatitis E virus (HEV), a major cause of viral hepatitis worldwide, is considered an emerging foodborne zoonosis in Europe. Pigs (*Sus scrofa domestica*) and wild boars (*S. scrofa*) are recognized as important HEV reservoirs. Additionally, HEV infection and exposure have been described in cervids. In Norway, HEV has been identified in pigs and humans; however, little is known regarding its presence in wild ungulates in the country. We used a species-independent double-antigen sandwich ELISA to detect antibodies against HEV in the sera of 715 wild ungulates from Norway, including 164 moose (*Alces alces*), 186 wild Eurasian tundra reindeer (*Rangifer tarandus tarandus*), 177 red deer (*Cervus elaphus*), 86 European roe deer (*Capreolus capreolus*), and 102 muskoxen (*Ovibos moschatus*). The overall seroprevalence was 12.3% (88/715). Wild reindeer had the highest seropositivity (23.1%, 43/186), followed by moose (19.5%, 32/164), muskoxen (5.9%, 6/102), and red deer (4%, 7/177). All roe deer were negative. According to our results, HEV is circulating in wild ungulates in Norway. The high seroprevalence observed in wild reindeer and moose indicates that these species may be potential reservoirs of HEV. To the authors’ knowledge, this is the first report of HEV exposure in reindeer from Europe and in muskoxen worldwide.

## 1. Introduction

Hepatitis E is one of the main causes of viral hepatitis worldwide [1] and is considered a major global public health problem [2]. The disease is caused by the hepatitis E virus (HEV), a positive-sense single-stranded RNA virus of the genus *Orthohepevirus*, family *Hepeviridae*, observed as non-enveloped particles in the feces and bile and as quasi-enveloped virions (covered by host lipids) in the serum [3,4]. This family contains two genera, *Piscihepevirus* and *Orthohepevirus*; the latter is able to affect birds and mammals [5] and comprises four recognized species (*Orthohepevirus A*, *B*, *C*, and *D*). At least eight genotypes of *Orthoherpevirus A* (syn. Hepatitis E virus) have been described. Genotypes 1 and 2 affect primates (mainly humans) and cause epidemics in developing countries (usually waterborne), with reports of genotype 1 infection in four horses from Egypt (interestingly, PCR-positive individuals presented with a significant elevation of aspartate aminotransferase when compared to PCR-negative horses) [5,6,7,8]. Genotypes 3 and 4 affect a broader range of mammalian hosts (e.g., pigs, wild boars, cervids, and humans) and are considered emerging foodborne zoonotic agents in developing and industrialized countries [5,7,9,10]. Genotypes 5 and 6 have been described only in wild boars (*Sus scrofa*) in Asia [11,12], genotype 7 in dromedaries (*Camelus dromedarius*) of the Arabian Peninsula [13] and in a liver transplant patient with chronic hepatitis [14], and genotype 8 was detected in Bactrian camels (*Camelus bactrianus*) from China [15].

Overall, the annual number of human HEV infections is estimated at approximately 20 million, leading to 3.3 million cases of symptomatic hepatitis and 44,000 deaths in 2015 alone [2]. In Europe, most human HEV infections are caused by genotype 3; however, local infection by genotype 4, as well as sporadic imported cases caused by genotypes 1, 2, and 4, have also been described [16]. A total of 21,018 confirmed hepatitis E cases were reported by 22 countries of the European Union/European Economic Area between 2005 and 2015 (although case definition varies by country), increasing almost yearly during the studied period, reaching a total of 28 fatal cases registered in five countries [17]. There are several reports of HEV exposure and infection in Fennoscandia (Finland, Sweden, Norway, and Russia), especially from Finland and Sweden [18,19,20,21].

A scientific opinion from the European Food Safety Authority in 2017 [22] on the public health risks associated with hepatitis E virus (HEV) as a foodborne pathogen highlighted the importance of both wild boar and deer meat in the transmission of HEV and provided strong advice to minimize the risk: “In order to minimize the risk of an HEV infection, consumers should thoroughly cook meat and offal, especially pork, wild boar and deer meat products”.

In Norway, HEV has been suggested as a neglected disease [23], not monitored by the Norwegian Surveillance System for Communicable Diseases. Information about the occurrence of HEV and its characteristics in the country is limited. To date, there are reports of three cases of HEV acute hepatitis presumably imported from Asia [24], HEV antibodies in a patient with acute hepatitis [25], and a serological survey description of anti-HEV IgG seroprevalence in veterinarians (13%, 21/163), blood donors (14%, 162/1200), and farm workers (30%, 24/79) from Norway [26]. Recently, an HEV seroprevalence of 11.4% (205/1800) was reported in people from Tromsø municipality, Northern Norway [27]. In animals, the only study performed in Norway found an elevated IgG-seroprevalence (73%, 484/663) in pigs [26]. Finally, Myrmel et al. [28] detected genotype 3 in Norwegian sewage plants.

Considering that most HEV infections in Europe are caused by zoonotic genotypes and that deer meat (raw or unprocessed) can represent a risk for public health, the goal of this study was to survey the seroprevalence of this virus in wild ungulates from Norway, in order to further understand its epidemiology, including potential reservoir species.

## 2. Materials and Methods

### 2.1. Samples

Overall, 715 ungulate serum samples were analyzed (Table 1): Eurasian tundra reindeer (*Rangifer tarandus tarandus*, n = 186), moose (*Alces alces*, n = 164), red deer (*Cervus elaphus*, n = 177), European roe deer (*Capreolus capreolus*, n = 86), and muskoxen (*Ovibos moschatus*, n = 102). Detailed information about the location (county of origin (Agder, Innlandet, Møre og Romsdal, Nordland, Oslo, Rogaland, Troms og Finnmark, Trøndelag, Vestfold og Telemark, Vestland, Viken, unknown)), age class (calf, juvenile, adult, unknown), sex (male, female, unknown), and sampling season (winter, spring, summer, autumn, unknown) of the tested species are displayed in Table 1. The samples were obtained between 2010 and 2018 in Norway. The cervid samples were collected during live animal captures for GPS-collaring projects in accordance with standard procedures for chemical immobilization of wild cervids [29]. All animals were immediately released post-collection. The muskoxen samples were collected from muskoxen killed by traffic accidents and governmental culling in Dovrefjell National Park as part of the species management plan. All protocols and licenses required for this study were approved by the Norwegian Environmental Agency (NEA) and the Norwegian Food Safety Authority (NFSA), responsible for enforcing regulations and issuing permits for the biological sampling of wild animals in Norway. As part of the national health surveillance of wildlife in Norway, NEA requires blood samples from all free-ranging cervids captured for scientific purposes to be submitted to the Norwegian Veterinary Institute (NVI). All blood samples used in our study were part of the NVI blood bank; therefore, the authors had no access to their original individual capture projects’ descriptions and field permit numbers. In order to separate the serum from blood cells, blood samples were collected in EDTA tubes and centrifuged at 3500 rpm for 5 min. Subsequently, the sera were transferred to 5 mL tubes and stored at −80 °C until testing.

### 2.2. Serological Study

We tested serum samples from 715 wild ungulates collected in Norway for the presence of antibodies (IgG, IgM, and IgA) against hepatitis E virus using a double antigen sandwich multispecies enzyme-linked immunosorbent assay (ELISA), HEV ELISA 4.0v Kit (MP Diagnostics, Illkirch, France), in accordance with the manufacturer’s instructions. This kit relies on the recombinant ORF2.1 antigen of the capsid protein, highly conserved in the different HEV genotypes, and has a sensitivity and specificity of 99.2% according to the manufacturer. This kit was previously used in cervids and other ungulates [30]. All serum samples were diluted in working conjugate at 1/5 dilution. The optical density (OD) was measured in a Labsystem Multiskan EX spectrophotometer at 450 nm (Thermo Electron Corporation, Waltham, MA, USA). OD values were correlated to a positive control serum following the manufacturer’s instruction. HEV positive and negative sera provided with the kit were used as positive and negative controls in each plate, respectively. HEV-seropositive serum samples from pigs were also used to monitor variations between the assays. All positive reactors were re-tested before they were conclusively identified as positive.

### 2.3. Statistics

Differences between the variables: species, sex, age class, as well as county, season, and year of sampling both for each species studied and for the whole database have been analyzed through a non-parametric test, Kruskall–Wallis, and Mann–Whitney U test as corresponding. All statistical analyses were performed in R studio software 3.0.1 [31] with a significance level of *p* < 0.05.

## 3. Results

Anti-HEV specific antibodies were detected in 88 animals of four different species by sandwich ELISA, including 23.1% (43/186) of Eurasian tundra reindeer, 19.5% (32/164) of moose, 5.9% (6/102) of muskoxen, and 4% (7/177) of red deer. All roe deer (n = 86) were seronegative. The results according to age (adult, juvenile, calf), sex (male, female), county of origin, and season (spring, summer, autumn, winter) of seropositive Eurasian tundra reindeer, moose, muskoxen, and red deer are shown in Supplementary Tables S1–S4, respectively. The overall hepatitis E virus seroprevalence results of the tested ungulates according to season and county of origin are recorded in Supplementary Table S5.

**Figure 1:**
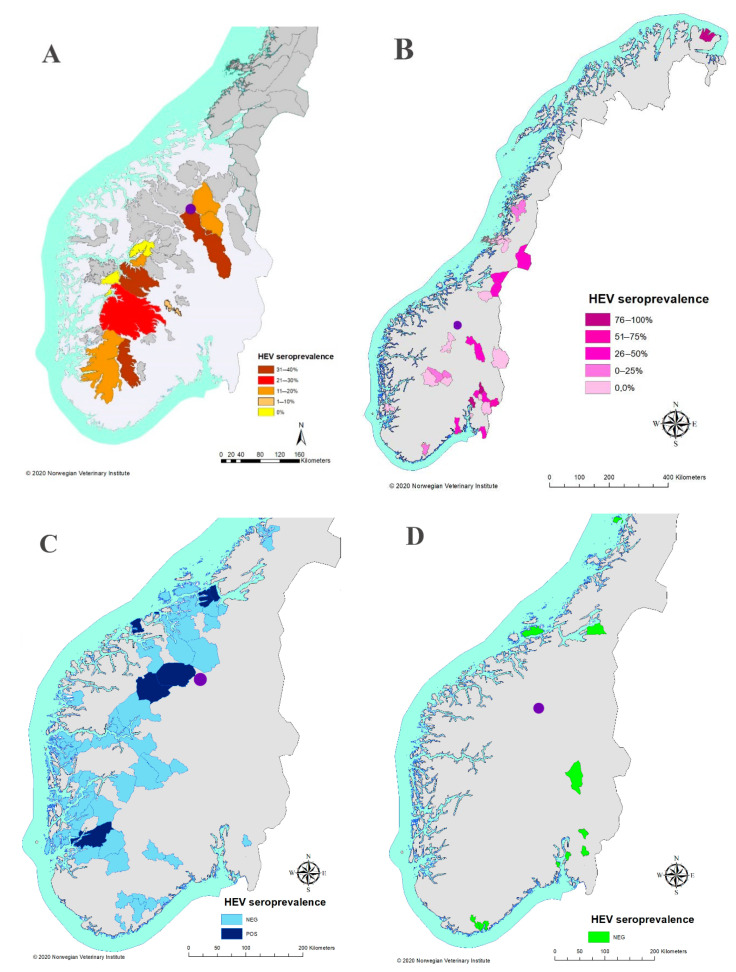
Overview of the origin of samples and prevalence results for Hepatitis E in this study. (**A**) Eurasian tundra reindeer (*Rangifer tarandus*); (**B**) moose (*Alces alces*); (**C**) red deer (*Cervus elaphus*); (**D**) European roe deer (*Capreolus capreolus*). All muskoxen (*Ovibos moschatus*) originated from Dovrefjell National Park, and this area is identified with a purple circle on every map. For information on the geographic units used on the maps, please refer to a higher format version of these in the Supplementary Materials (Supplementary Figure S1A–D). Note that for some cases, the geographical origin was not recorded; therefore, the total number of cases does not necessarily coincide with the number of cases represented.

The geographic origin of seropositive and seronegative-tested animals is shown in Figure 1. Statistically significant differences in HEV prevalence between species were found (*p =* 0.001, *K* = 55.36). Specifically, significantly higher HEV prevalence values were noted in reindeer when compared to muskoxen (*p* = 0.002) and red deer (*p* = 0.0001), in moose when compared to muskoxen (*p* = 0.0021) and red deer (*p* = 0.0001), and in reindeer and moose when compared to roe deer (both with values of *p* = 0.0001).

**Figure 2:**
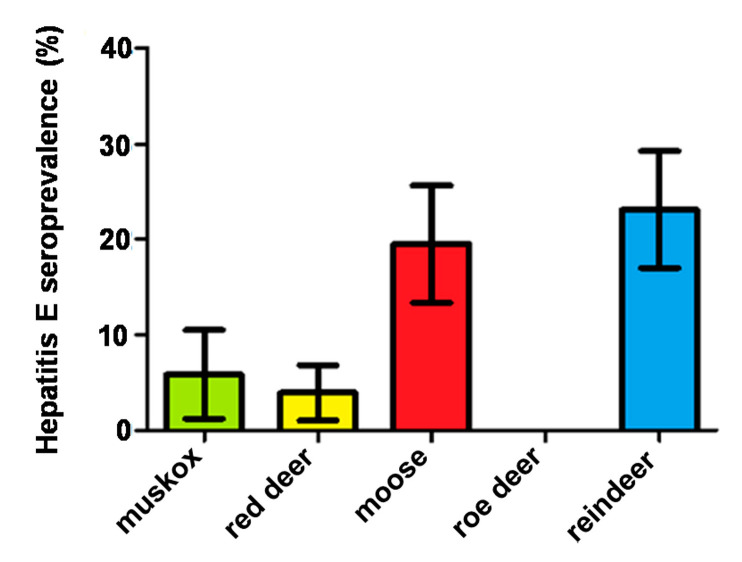
Seroprevalence values for the tested muskoxen (*Ovibos moschatus*), red deer (*Cervus elaphus*), moose (*Alces alces*), roe deer (*Capreolus capreolus*), and European Eurasian tundra reindeer (*Rangifer tarandus*) evaluated in the study. The 95% confidence interval is represented in the T-shaped bars.

Analyzing each of the species independently, no differences in HEV prevalence were found between sexes, age classes, origin, season, or year of sampling (Supplementary Tables S1–S4, Figure 2). Likewise, analyzing the whole database altogether, no differences in HEV prevalence were found with respect to season, origin of the samples, or year of sampling (Supplementary Table S5).

## 4. Discussion

Present results showed significantly high HEV seroprevalences in reindeer and moose when compared to the remaining species. Anti-HEV antibodies were also found in red deer and muskoxen. All roe deer were seronegative. To the authors’ knowledge, these are the first data regarding the seroprevalence of HEV in wild ungulates from Norway as well as the first data on reindeer from Europe and muskoxen worldwide. Our results indicate that Hepatitis E viruses are circulating in wild ungulates (cervids and muskoxen) and that the infection is apparently endemic in cervids in Norway since seropositive samples were detected in almost every studied year (2010–2018) and most of the studied regions (in all the counties of sampling but Rogaland). The high seroprevalence values observed in reindeer and moose suggest that these species may act as reservoirs, playing an important role in the epidemiology of the disease caused by zoonotic genotypes in Norway; however, it is not possible to exclude their infection in spillover events. One cannot also overlook the possibility of an unknown specific cervid hepatitis E virus, antigenically closely related to those causing disease in humans, being detected by the ELISA used in this study. Thus, future molecular studies are necessary to characterize the viruses present in these species in order to clarify the epidemiological role played by reindeer and moose in Norway. In other countries, suids (domestic pigs and wild boars) have been identified as one of the most important domestic and wildlife HEV reservoirs for human infection by zoonotic genotypes [32]. Nevertheless, wild boars are almost completely absent in Norway, with only a small population of about 1000 individuals in the southeast (mainly in Østfold county) [33], and pig farming is somewhat limited (approximately 80,000 sows) [34]. Therefore, the contribution of suids to the circulation of HEV in Norway is probably moderate, suggesting that cervids could have a bigger role in the epidemiology of HEV in Norway. The circulation of HEV in red deer and chamois (*Rupicapra rupicapra*) in certain Alpine regions without suids has been described by Trogu et al. [35]. The low seroprevalence rates found in muskoxen and red deer (5.9%, 6/102, and 4%, 7/177, respectively) indicate that they are possibly incidental HEV hosts in Norway.

Unfortunately, there are no previous HEV studies in wild or semi-domestic reindeer from Europe available for comparison. However, 25 serum samples of woodland caribou (*Rangifer tarandus caribou*) and porcupine caribou (*R. tarandus granti*) from Canada tested seronegative for HEV [36], while Weger et al. [37] detected seroprevalence rates of 1.7 (n = 2/120) in barren-ground caribou (*R. tarandus groenlandicus*) and 5.2% (5/97) woodland caribou, also from Canada. The prevalence rates observed in Canadian caribous are much lower than those of Eurasian tundra reindeer from Norway (23.1%, 43/186) observed in the present study.

In moose, Lin et al. [38] reported a divergent HEV type with unknown zoonotic potential. That strain was subsequently identified in twelve moose serum and/or stool samples from Sweden, but not in humans or wild boars from that country [39]. Therefore, moose have been proposed as the only host for moose hepatitis [39]. The moose HEV-seroprevalence rate observed in Norway (19.5%, 32/164) is higher than those previously found in moose from Sweden (14%, 9/66) [39], Finland (9.1%, 31/342) [40], and Lithuania (11.8, 4/34) [41]. Given the high prevalence in Norway, and for matters of public health, it will be important to investigate in the future if moose in Norway are infected with the known zoonotic HEV genotypes or a specific moose hepatitis E virus as found in Sweden.

In red deer, the seroprevalence found in the present study (4%, 7/177) is similar to the one described in the Netherlands (5%, 2/38) by Rutjes et al. [42]. However, higher values have been observed in red deer from Spain (10.4%, 101/968 to 12.85%, 9/70), Italy (13.9%, 35/251), and Sweden (7%, 1/14) [39,43,44,45]. The HEV seroprevalence in red deer appears to be generally low in Europe, as reported in Germany (rates of 2%, 2/100 and 3.3%, 2/61) [46], Belgium (1%, 2/189) [30], and Italy (0.8%, 2/254 to 2.6%, 1/38) [35,47]. All red deer studied in Poland (n = 118) and in Germany (n = 78) were seronegative [48,49].

The lack of anti-HEV antibodies in Norwegian roe deer resembled the findings from surveys in eight roe deer tested in the Netherlands [42], 59 roe deer from Germany [49], 38 roe deer from Poland [48], and 12 roe deer from Finland [40]—all of them HEV-seronegative. By contrast, HEV exposure was described in roe deer from Sweden (7%, 2/29) [39], different regions of Germany (5.4–6.8% (2/37 and 8/117, respectively)) [46], Italy (3.1%, 1/32) [47], and Belgium (3%, 7/235) [30]. To the authors’ knowledge, only zoonotic genotype 3 infections have been described in roe and red deer [41,44,49,50].

Muskoxen are one of the largest members of the subfamily Caprinae (family Bovidae), along with the takins (*Budorcas taxicolor*) [51]. There are no previous HEV reports in muskoxen for comparison. Interestingly, in recent years, HEV has been serologically and molecularly reported in domestic Caprinae, with 21.3% (41/192) seroprevalence and 10.4% (20/192) of genotype 3 detection rate in sheep from Southern Italy [32], 21.6% (29/134) seroprevalence and 3% (4/134) real-time RT-PCR prevalence in sheep from Northern Italy [47], and 35.2% (176/500) seroprevalence and 5.3% (4/75) genotype 4 detection rate in sheep from China [52]. Additionally, genotype 3 was also detected in goats from Italy (9.2%, 11/119) [53] and also HEV exposure (11.4%, 19/167) [47]. Regarding wild Caprinae, HEV-exposure was recently described in chamois (*Rupicapra rupicapra*) [35] and Alpine ibex (*Capra ibex*) [47] from Italy (seroprevalences of 1.2% [2/172] and 6.3% [2/32], respectively).

When comparing different seroprevalence values, it is important to consider the particular characteristics of each serological method employed and the study design, most of them based on convenience sampling.

The consumption of undercooked or raw meat or viscera and untreated milk and derivates of ungulates represents a potential risk for human health regarding infection by the zoonotic HEV genotypes as highlighted by EFSA in 2017 [22]. Most zoonotic cases have been linked to the consumption of pig and wild boar products [54]. Nevertheless, consumption of deer products is also considered a risk factor [55], with genotype 3 and 4 infections in humans following the ingesting of raw roe deer meat [55,56]. Additionally, a likely zoonotic-genotype 7 infection was identified in a man presenting with hepatitis and a history of regular consumption of camel meat and milk [14]. Interestingly, experimental oral inoculation of genotype 4 HEV in raw and pasteurized cow milk was infectious for rhesus macaques (*Macaca mulatta*) [57], suggesting that this product may have an underestimated role in hepatitis E transmission.

Although cervids are socioeconomically and culturally important in Norway [58], the potential zoonotic threat of consuming their meat, viscera, or milk has not been assessed. Moose, red deer, and roe deer are important game species in the country, sustaining very large populations [59,60,61], and their meat is directed to human consumption [58]. Two different populations of reindeer are present in Norway: the last free-ranging tundra reindeer from Europe in the south and a large semi-domestic population in the north. The latter is an important resource for the Sami people, the only indigenous people from Scandinavia. Although traditional reindeer milking is rarely practiced in the Sami culture today, reindeer milk and derivates are still sold for human consumption, mainly destined for the tourism sector [62]. Reindeer meat (including dry and smoked meat) is an important part of the Sami diet [63] and is often consumed in Northern Norway [64]. In contrast to the studied cervids, muskox is a reintroduced species in Norway, with a small current population of approximately 300 individuals, restricted to the Dovrefjell National Park [65]. The high reindeer and moose HEV seroprevalence observed in this study indicates that HEV-foodborne transmission through cervid products should be further investigated.

The pathogenicity of HEV, as well as its associated lesions, in cervids and muskoxen remains unknown. In humans, genotypes 1 and 2 are generally associated with severe acute hepatitis, including fulminant hepatitis in pregnant women, while genotypes 3 and 4 can cause acute or chronic hepatitis in immunocompromised hosts [66,67]. Additionally, genotype 7 was described in an immunocompromised man who developed chronic hepatitis [14]. In HEV-positive pigs, necroinflammatory hepatic lesions similar to those observed in human cases have been reported by histopathology [68]. Given this study’s results in Norwegian reindeer and moose in Norway, we believe that wildlife necropsies should include HEV in their differentials when hepatic lesions are observed. This may represent valuable information to further understand the clinical implications of HEV in these species and help to characterize the circulating HEV genotypes.

## 5. Conclusions

Herein, we provide the first data regarding HEV exposure in wildlife from Norway, including the first report on reindeer from Europe and in muskoxen worldwide. The high seroprevalence observed in reindeer and moose suggests a potential role as reservoir species. Additionally, cervids and muskoxen may be useful sentinels for HEV monitoring. However, further molecular investigations are needed to demonstrate the presence of HEV in the wild ungulate species sampled and in order to better evaluate their epidemiological role in the transmission and maintenance of infection. A One Health approach, with the collaboration of physicians, farmers, and domestic and wildlife veterinarians, is necessary to elucidate the epidemiology of hepatitis E and provide the tools for its control. Further investigations are warranted to address the molecular identification of the HEV genotype(s) infecting ungulate species of Norway and possible HEV-associated lesions. The inclusion of HEV in the ongoing wildlife surveillance program in Norway is also advised.

## Figures and Tables

**Table 1:** Age class (C = calf, J = juvenile, A = adult, U = unknown) and sex (M = male, F = female, U = unknown) of the tested moose (*Alces alces*), red deer (*Cervus elaphus*), European roe deer (*Capreolus capreolus*), Eurasian tundra reindeer (*Rangifer tarandus*), and muskoxen (*Ovibos moschatus*) included in the study, and season of sampling (W = winter, Sp = spring, S = summer, A = Autumn, NR = not recorded).

Species	County	Nº of Animals	HEV Positive Animals	Age Class				Sex			Season				
				C	J	A	U	M	F	U	W	Sp	S	A	NR
**Reindeer**	Agder	41	8	-	-	31	10	5	34	2	30	8	1	2	-
	Innlandet	50	12	-	4	43	3	4	40	6	41	3	6	-	-
	Vestfold og Telemark	9	3	-	-	9	-	-	9	-	-	9	-	-	-
	Vestland	60	12	-	12	41	7	7	53	-	36	24	-	-	-
	Viken	6	1	-	1	4	1	-	6	-	-	6	-	-	-
	Not recorded	20	7	-	9	11	-	12	8	-	9	11	-	-	-
	***subtotal***	***186***	***43***	***0***	***26***	***139***	***21***	***28***	***150***	***8***	***116***	***61***	***7***	***2***	***0***
**Moose**	Agder	4	1	-	2	2	-	3	1	-	-	-	-	4	-
	Innlandet	34	9	7	5	22	-	11	21	2	13	-	-	20	1
	Nordland	21	1	4	3	9	5	5	16	-	16	-	-	4	1
	Oslo	1	1	-	-	1	-	1	-	-	1	-	-	-	-
	Rogaland	1	-	-	1	-	-	-	-	1	-	-	1	-	-
	Troms og Finnmark	11	1	-	-	11	-	1	10	-	4	7	-	-	-
	Trøndelag	33	4	12	5	16	-	13	17	3	22	0	0	10	1
	Vestfold og Telemark	12	2	4	3	4	1	9	3	-	-	-	-	10	2
	Viken	47	13	5	7	35	-	13	33	1	34	-	-	13	-
	***subtotal***	***164***	***32***	***32***	***26***	***100***	***6***	***56***	***101***	***7***	***90***	***7***	***1***	***61***	***5***
**Red deer**	Agder	15	-	-	-	3	12	3	12	-	15	-	-	-	-
	Innlandet	16	2	2	2	12	-	11	5	-	-	-	5	11	-
	Møre og Romsdal	20	2	1	1	12	6	9	11	-	17	3	-	-	-
	Rogaland	17	1	6	-	10	1	6	11	-	10	6	-	1	-
	Trøndelag	33	2	5	4	20	4	18	15	-	15	8	7	3	-
	Vestfold og Telemark	11	-	-	-	5	6	3	8	-	11	-	-	-	-
	Vestland	44	-	2	1	24	17	12	30	2	25	14	-	4	1
	Viken	21	-	-	-	-	21	-	20	1	20	-		-	1
	***subtotal***	***177***	***7***	***16***	***8***	***86***	***67***	***62***	***112***	***3***	***113***	***31***	***12***	***19***	***2***
**Roe deer**	Agder	9	-	1	1	7	-	7	2	-	-	-	1	5	3
	Innlandet	10	-	2	2	6	-	6	4	-	-	-	-	10	-
	Nordland	16	-	7	1	8	-	6	10	-	-	-	-	14	2
	Trøndelag	22	-	7	2	13	-	15	7	-	-	-	7	14	1
	Vestfold og Telemark	9	-	5	1	3	-	6	3	-	-	-	-	8	1
	Viken	20	-	2	6	12	-	15	5	-	-	-	4	13	3
	***Subtotal***	***86***	***0***	***24***	***13***	***49***	***0***	***55***	***31***	***0***	***0***	***0***	***12***	***64***	***10***
**Muskox**	***Dovrefjell NP subtotal***	***102***	***6***	***24***	***13***	***65***	***0***	***56***	***46***	***0***	***12***	***39***	***21***	***1***	***29***
	**Total**	**715**	**88**	**96**	**86**	**439**	**94**	**257**	**440**	**18**	**331**	**138**	**53**	**147**	**46**

## Data Availability

All data are available in the manuscript.

## Supplementary Materials

The following are available online at https://www.mdpi.com/1999-4915/13/2/224/s1, Figure S1A: Overview of the origin and prevalence results of samples tested for HEV in this study for Eurasian tundra reindeer (Rangifer tarandus). Figure S1B: Overview of the origin and prevalence results of samples tested for HEV in this study for moose (Alces alces). Figure S1C: Overview of the origin and prevalence results of samples tested for HEV in this study for red deer (Cervus elaphus). Figure S1D: Overview of the origin and prevalence results of samples tested for HEV in this study for European roe deer (Capreolus capreolus), Table S1: Seroprevalence of hepatitis E virus and statistical results in the tested reindeer (Rangifer tarandus) from Norway according to season age (adult, juvenile), sex (male, female), county of origin (Agder, Innlandet, Vestfold, Vestland, Viken) and season (spring, summer, autumn, winter). Table S2: Seroprevalence of hepatitis E virus and statistical results in the tested moose (Alces alces) from Norway according to season age (adult, subadult, calf), sex (male, female), county of origin (Agder, Innlandet, Nordland, Oslo, Rogland, Troms og Finnmark, Trondelag, Vestfold, Viken) and season (spring, summer, autumn, winter). Table S3: Seroprevalence of hepatitis E virus and statistical results in the tested muskoxen (Ovibos moschatus) from Norway according to season age (adult, subadult, calf), sex (male, female), and season (spring, summer, autumn, winter). All the animals were sampled in the Dovrefjell National Park. Table S4: Seroprevalence of hepatitis E virus and statistical results in the tested red deer (Cervus elaphus) from Norway according to season age (adult, subadult, calf), sex (male, female), county of origin (Agder, Innlandet, Møre og Romsdal, Rogland, Trondelag, Vestfold, Vestland, Viken) and season (spring, summer, autumn, winter). Table S5: Seroprevalence of hepatitis E virus and statistical results in the tested ungulate species from Norway according to season (spring, summer, autumn, winter) and county of origin (Agder, Innlandet, Møre og Romsdal, Nordland, Oslo, Rogland, Troms og Finnmark, Trondelag, Vestfold, Vestland, Viken).
