# Supplementary material for: Seroprevalence of Hepatitis E Virus in Moose (Alces alces), Reindeer (Rangifer tarandus), Red Deer (Cervus elaphus), Roe Deer (Capreolus capreolus), and Muskoxen (Ovibos moschatus) from Norway
Source: Viruses. 2021 Feb 1;13(2):224. doi: 10.3390/v13020224 (PMC7912786; doi:10.3390/v13020224)
Supplement: Supplementary file 1 [file viruses-13-00224-s001.pdf]

viruses

MDPI

Supplementary Material

Table S1. Seroprevalence of hepatitis E virus and statistical results in the tested reindeer (*Rangifer tarandus*) from Norway according to season age (adult, juvenile), sex (male, female), county of origin (Agder, Innlandet, Vestfold, Vestland, Viken) and season (spring, summer, autumn, winter).

	**Age [1]21**		**Sex [1]8**		**County [1]61**					**Season [1]2**			
	**Adult**	**Juvenile**	**Male**	**Female**	**Agder**	**Innlandet**	**Vestfold**	**Vestland**	**Viken**	**Spring**	**Summer**	**Autumn**	**Winter**
N	139	26	28	150	41	50	9	60	6	61	7	0.0	116
Prevalence %	25.0	12.0	25.0	24.0	20.0	24.0	33.0	20.0	17.0	20.0	29.0	0.0	25.0
95% CI	18.0–32.0	−16.0–25.0	7.9–42	17.0–31.0	6.8–32.0	12–36.0	−5.1–72.0	9.6–30.0	−26.0–60.0	9.4–30	−17.0–74.0	0.0	17.0–33.0
p-value	0.13		0.91		0.88					0.69		-	
Statistic value *K* or *U* *as corresponding	1561		2079		1173					0.74			

[1] Number of animals not- analyzed for this variable.

Table S2. Seroprevalence of hepatitis E virus and statistical results in the tested moose (*Alces alces*) from Norway according to season age (adult, subadult, calf), sex (male, female), county of origin (Agder, Innlandet, Nordland, Oslo, Rogaland, Troms og Finnmark, Trondelag, Vestfold, Viken) and season (spring, summer, autumn, winter).

	**Age [1]6**			**Sex [1]7**		**County [1]4**									**Season [1]5**			
	**Adult**	**Juvenile**	**Calf**	**Male**	**Female**	**Agder**	**Innlandet**	**Nordland**	**Oslo**	**Rogaland**	**Troms**	**Trøndelag**	**Vestfold**	**Viken**	**Spring**	**Summer**	**Autumn**	**Winter**
N	100	26	32	56	101	4	34	21	1	1	11	33	12	47	7	1	61	90
Prevalence %	26.0	7.7	13.0	23.0	19.0	25.0	26.0	4.8	100	0.0	9.1	12.0	17.0	28.0	14.0	0.0	21.0	18.0
95% CI	17.0–35	−3.3–19	0.4–25	12.0–35	11.0–27	−55–100	11–42	−5.2–15	0.0–0.0	0.0–0.0	−0.11	0.4–24	−8.1–41	14–41	−21–49	0.0–0.0	11.0–32	9.7–26
p-value	0.06			0.5		0.1									0.9			
Statistic value *K* or *U* *as corresponding	5.7			2704		12.3									0.6			

[1] Number of animals not- analyzed for this variable.

Table S3. Seroprevalence of hepatitis E virus and statistical results in the tested muskoxen (*Ovibos moschatus*) from Norway according to season age (adult, subadult, calf), sex (male, female), and season (spring, summer, autumn, winter). All the animals were sampled in the Dovrefjell National Park.

	**Age[1]0**			**Sex[1]9**		**Season [1]43**			
	**Adult**	**Juvenile**	**Calf**	**Male**	**Female**	**Spring**	**Summer**	**Autumn**	**Winter**
N	65	13	24	47	46	39	10	1	9
Prevalence %	7.7	7.7	0.0	4.3	8.7	10.0	0.0	0.0	0.0
95% CI	1.0–14	−9.1–24	0.0–0.0	−1.7–10	0.2–17	0.3–20	0.0–0.0	0.0–0.0	0.0–0.0
p-value		0.38		0.39			0.54		
Statistic value *K* or *U* *as corresponding		1.94		1033			2.1		

[1] Number of animals not- analyzed for this variable.

Table S4. Seroprevalence of hepatitis E virus and statistical results in the tested red deer (*Cervus elaphus*) from Norway according to season age (adult, subadult, calf), sex (male, female), county of origin (Agder, Innlandet, Møre og Romsdal, Rogland, Trondelag, Vestfold, Vestland, Viken) and season (spring, summer, autumn, winter).

	**Age[1]102**			**Sex[1]47**		**County[1]0**								**Season[1]2**			
	**Adult**	**Juvenile**	**Calf**	**Male**	**Female**	**Agder**	**Innlandet**	**Møre og Romsdal**	**Rogaland**	**Trøndelag**	**Vestfold**	**Vestland**	**Viken**	**Spring**	**Summer**	**Autumn**	**Winter**
N	50	9	16	62	68	15	16	20	17	33	11	44	21	31	12	19	113
Prevalence %	6	0.0	0.0	4.8	2.9	0	13.0	10.0	5.9	6.1	0.0	0.0	0.0	0.0	8.3	5.3	4.4
95% CI	−0.8–13	0.0–0.0	0.0–0.0	−0.6–10	−1.2–7.1	0.0–0.0	−5.7–31	−4.4–24	−6.6–18	−2.5–15	0.0–0.0	0.0–0.0	0.0–0.0	0.0–0.0	−0.10	−5.8–6	0.6–8.3
p-value		0.46		0.58					0.24						0.57		
Statistic value *K* or *U* *as corresponding		1.5		2068					9.24						2.0		

[1]Number of animals not- analyzed for this variable.

Table S5. Seroprevalence of hepatitis E virus and statistical results in the tested ungulate species from Norway according to season (spring, summer, autumn, winter) and county of origin (Agder, Innlandet, Møre og Romsdal, Nordland, Oslo, Rogland, Troms og Finnmark, Trondelag, Vestfold, Vestland, Viken).

	**Season**				**County**										
	**Spring**	**Summer**	**Autumn**	**Winter**	**Agder**	**Innlandet**	**Møre og Romsdal**	**Nordland**	**Oslo**	**Rogland**	**Troms og Finnmark**	**Trondelag**	**Vestfold**	**Vestland**	**Viken**
N	137	53	145	331	69	158	29	37	1	18	11	116	41	104	94
Prevalence	12.0	5.7	9.7	15.0	13.0	17.0	6.9	2.7	100	5.6	9.1	6.0	12.0	12.0	15.0
95% CI	6.8–18	−0.7–12.0	4.8–15.0	11–19.0	4.9–21.2	11.0–23.0	−2.9–17.0	−2.8–8.2	0.0–0.0	−6.2–17.0	−11.0–29.0	1.6–10.0	1.7–23.0	5.3–18.0	7.6–22.0
p-value	0.13				0.15										
Statistic value *K*	5.7				13.4										

viruses

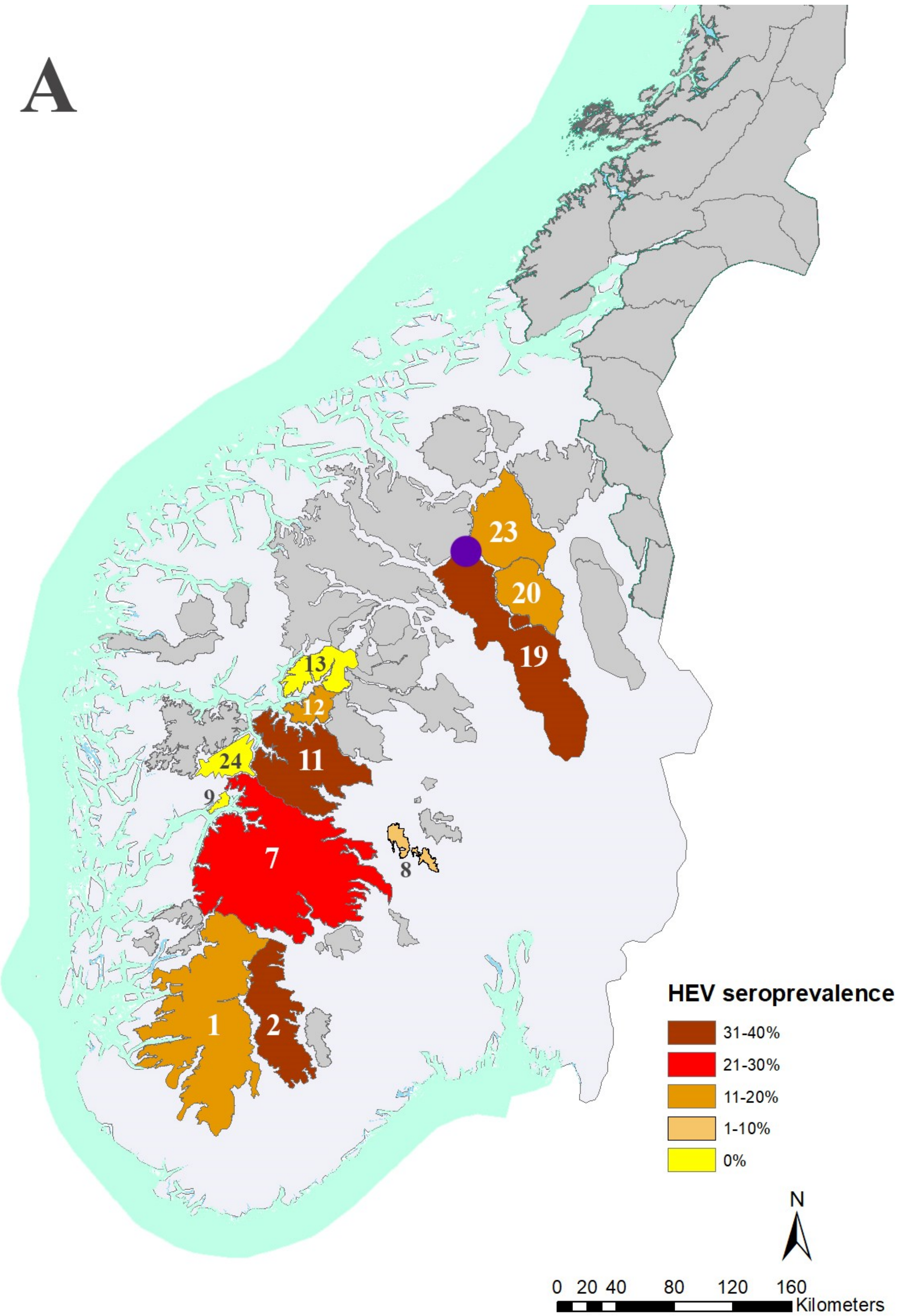

© 2020 Norwegian Veterinary Institute

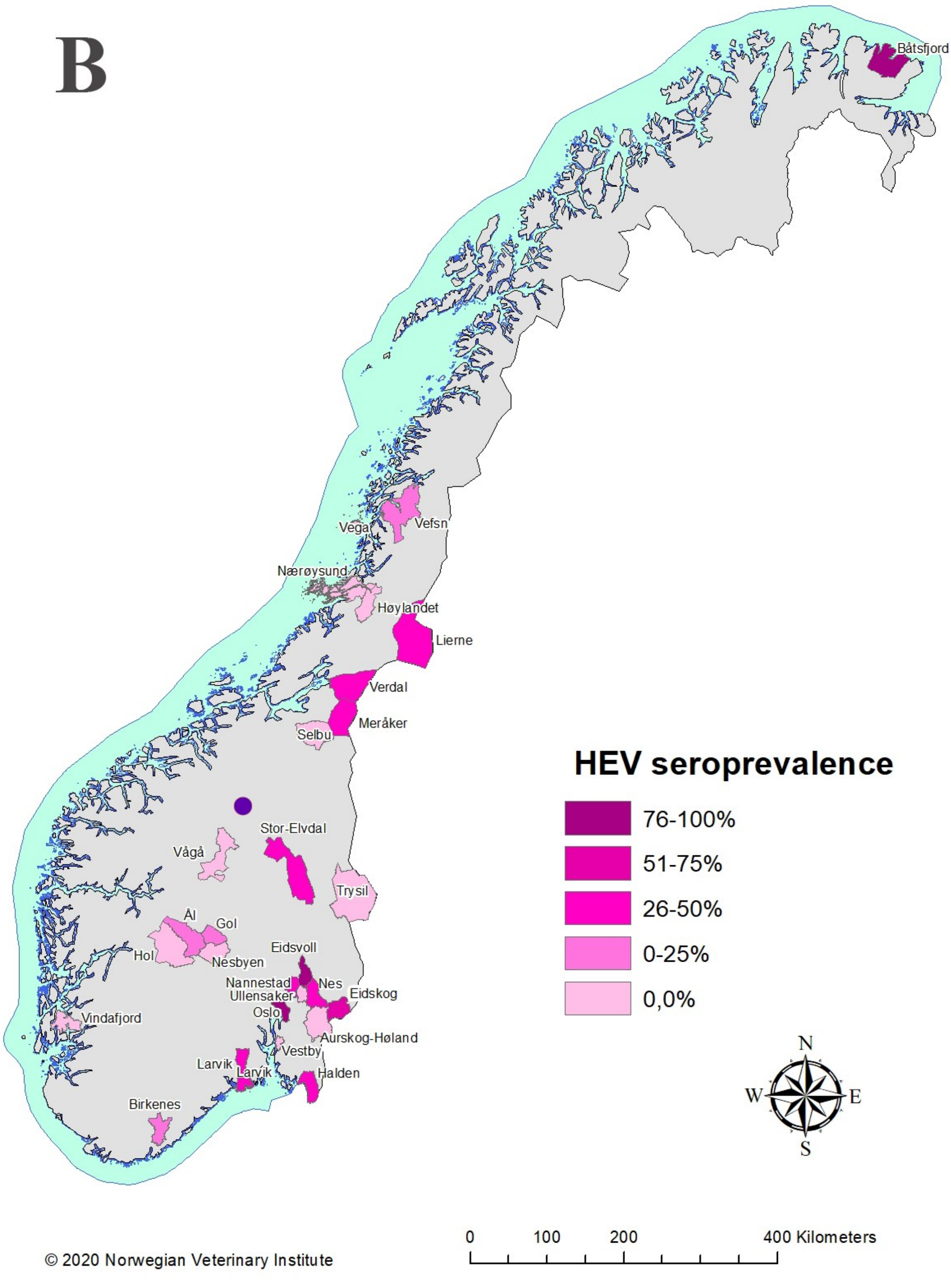

© 2020 Norwegian Veterinary Institute

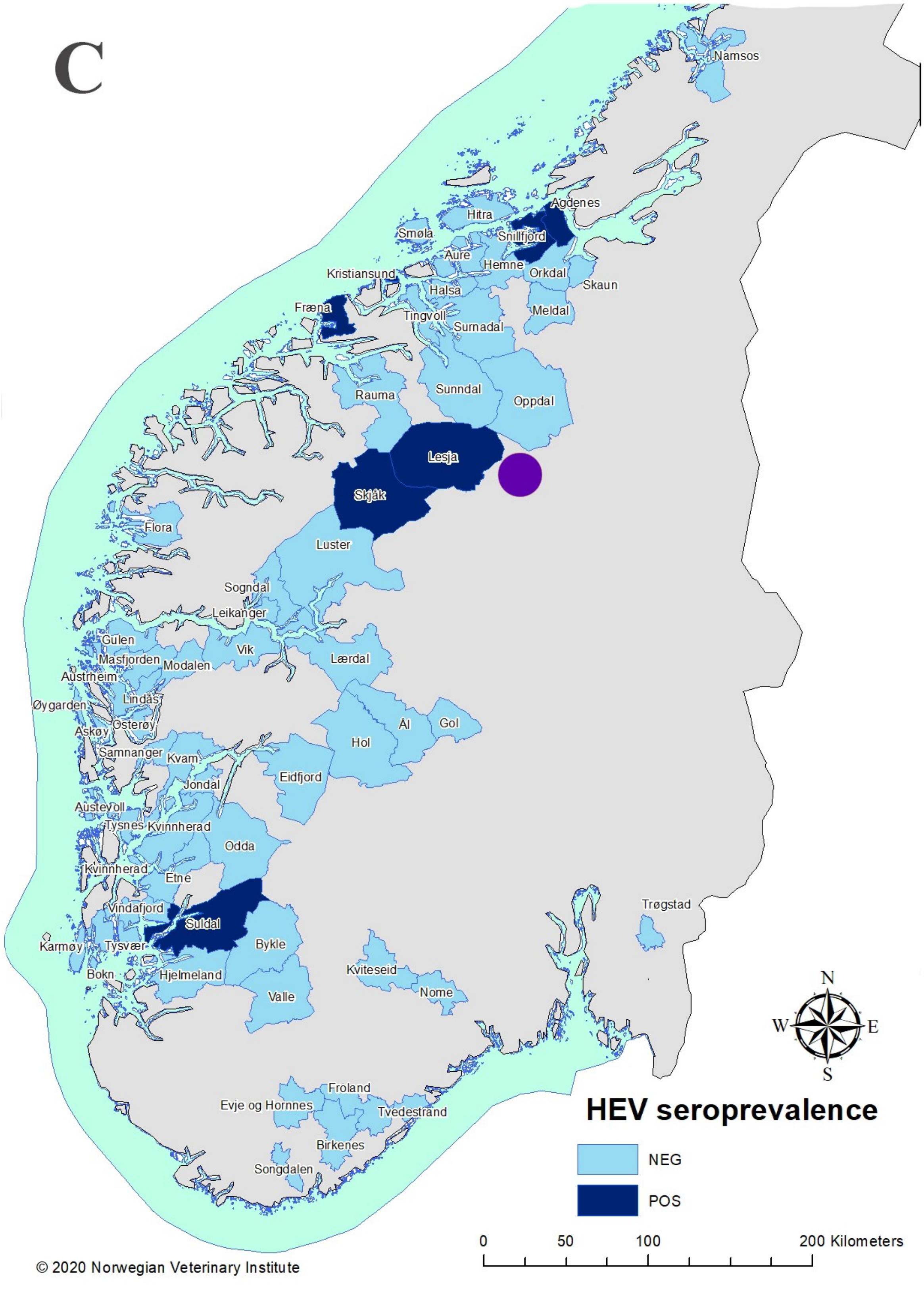

© 2020 Norwegian Veterinary Institute

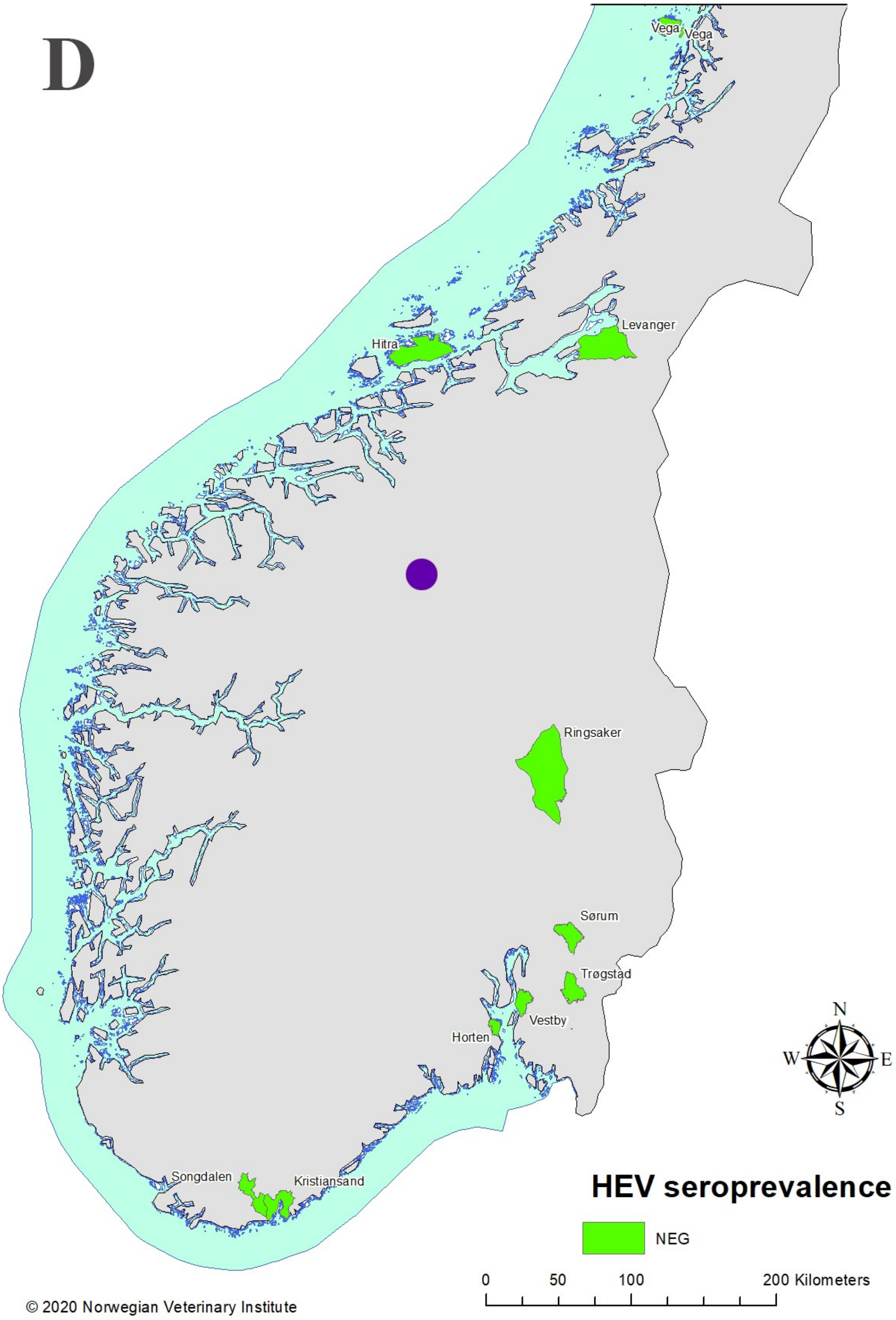

Figure S1. (**A**) Overview of the origin and prevalence results of samples tested for HEV in this study for Eurasian tundra reindeer (*Rangifer tarandus*). The numbers refer to the different wildlife reindeer management districts: 1-Setesdal Rykylke; 2-Setesdal Austhei; 7-Hardangervidda; 8-Norefjell; 9-Oksenhavøya; 11-Nordfjella; 12-Lærdal/Årdal; 13-Vest Jotunheimen; 19-Rondane; 20-Sølnkletten; 23-Knutshø; 24-Raudafjell. All muskoxen (*Ovibos moschatus*) originated from Dovrefjell National

Park, and this area is identified with a purple circle in every map. Note that for some cases the geographical origin was not recorded, therefore the total number of cases does not necessarily coincide with the number of cases represented. (**B**) Overview of the origin and prevalence results of samples tested for HEV in this study for moose (*Alces alces*). Geographic units colored in the map represent municipalities where animal samples were collected. All muskoxen (*Ovibos moschatus*) originated from Dovrefjell National Park, and this area is identified with a purple circle in every map. Note that for some cases the geographical origin was not recorded, therefore the total number of cases does not necessarily coincide with the number of cases represented. (**C**) Overview of the origin and prevalence results of samples tested for HEV in this study for red deer (*Cervus elaphus*). Geographic units colored in the map represent municipalities where animal samples were collected. All muskoxen (*Ovibos moschatus* originated from Dovrefjell National Park and this area is identified with a purple circle in every map. Note that for some cases the geographical origin was not recorded, therefore the total number of cases does not necessarily coincide with the number of cases represented. (**D**) Overview of the origin and prevalence results of samples tested for HEV in this study for European roe deer (*Capreolus capreolus*). Geographic units colored in the map represent municipalities where animal samples were collected. All muskoxen (*Ovibos moschatus*) originated from Dovrefjell National Park and this area is identified with a purple circle in every map. Note that for some cases the geographical origin was not recorded, therefore the total number of cases does not necessarily coincide with the number of cases represented.
